# Impact of the supramolecular structure of cellulose on the efficiency of enzymatic hydrolysis

**DOI:** 10.1186/s13068-015-0236-9

**Published:** 2015-04-01

**Authors:** Ausra Peciulyte, Katarina Karlström, Per Tomas Larsson, Lisbeth Olsson

**Affiliations:** Department of Biology and Biological Engineering, Division of Industrial Biotechnology, Chalmers University of Technology, Kemivägen 10, Gothenburg, SE-412 96 Sweden; Innventia AB, Drottning Kristinas väg 61, Stockholm, SE-114 86 Sweden; Wallenberg Wood Science Center, KTH Royal Institute of Technology, Teknikringen 56-58, Stockholm, SE-100 44 Sweden; Wallenberg Wood Science Center, Chalmers University of Technology, Kemigården 4, Gothenburg, SE-412 96 Sweden

**Keywords:** Cellulose I, Enzymatic hydrolysis, Cellulose supramolecular structure, Solid-state cross-polarization magic angle spinning carbon-13 nuclear magnetic resonance (CP/MAS ^13^C-NMR), Porosity, Crystallinity

## Abstract

**Background:**

The efficiency of enzymatic hydrolysis is reduced by the structural properties of cellulose. Although efforts have been made to explain the mechanism of enzymatic hydrolysis of cellulose by considering the interaction of cellulolytic enzymes with cellulose or the changes in the structure of cellulose during enzymatic hydrolysis, the process of cellulose hydrolysis is not yet fully understood. We have analysed the characteristics of the complex supramolecular structure of cellulose on the nanometre scale in terms of the spatial distribution of fibrils and fibril aggregates, the accessible surface area and the crystallinity during enzymatic hydrolysis. Influence of the porosity of the substrates and the hydrolysability was also investigated. All cellulosic substrates used in this study contained more than 96% cellulose.

**Results:**

Conversion yields of six cellulosic substrates were as follows, in descending order: nano-crystalline cellulose produced from never-dried soda pulp (NCC-OPHS-ND) > never-dried soda pulp (OPHS-ND) > dried soda pulp (OPHS-D) > Avicel > cotton treated with sodium hydroxide (cotton + NaOH) > cotton.

**Conclusions:**

No significant correlations were observed between the yield of conversion and supramolecular characteristics, such as specific surface area (SSA) and lateral fibril dimensions (LFD). A strong correlation was found between the average pore size of the starting material and the enzymatic conversion yield. The degree of crystallinity was maintained during enzymatic hydrolysis of the cellulosic substrates, contradicting previous explanations of the increasing crystallinity of cellulose during enzymatic hydrolysis. Both acid and enzymatic hydrolysis can increase the LFD, but no plausible mechanisms could be identified. The sample with the highest initial degree of crystallinity, NCC-OPHS-ND, exhibited the highest conversion yield, but this was not accompanied by any change in LFD, indicating that the hydrolysis mechanism is not based on lateral erosion.

**Electronic supplementary material:**

The online version of this article (doi:10.1186/s13068-015-0236-9) contains supplementary material, which is available to authorized users.

## Background

Cellulose, one of the most abundant organic materials on Earth, is found in raw materials such as forestry and agricultural residues and various kinds of waste. The enzymatic hydrolysis of cellulose is generally considered to be a sustainable means of obtaining monosaccharides that can be converted into a number of products via microbial fermentation [1]. Bioethanol is the prime example of the conversion of monosaccharides into renewable transportation fuels employing fermentation [2]. However, the enzymatic hydrolysis of cellulose is often incomplete [3] and we do not yet have a full understanding of the process.

The types of enzymes required for the enzymatic hydrolysis of cellulose include endocellulases, exocellulases and β-glucosidase [3-5]. A new enzyme family, AA9 (formerly GH61), harbouring fungal enzymes and which functions in the same way as lytic polysaccharide monooxygenases has recently been introduced [6,7]. Filamentous fungi, such as the thoroughly investigated *Trichoderma reesei*, and certain bacteria secrete cellulases (non-complexed) extracellularly. Also, multienzyme complexes (cellulosomes) are known to be produced by other bacteria. It has been shown that non-complexed fungal cellulases deconstruct plant cell walls using a different mechanism from the one used by cellulosomes [8]. The rate of enzymatic hydrolysis usually decreases in time. Various enzyme-related factors as possible causes have been discussed in the literature, such as: product inhibition [9]; enzyme deactivation due to heat, exposure to the air-liquid interface and/or mechanical factors [9]; overcrowding of bound cellulases on cellulose surface (called as traffic jams) causes interference between cellulases and reduces their hydrolytic efficiency [10]; non-productively bound cellulases [9]; loss of enzyme synergy [11] and competition between enzymes [12]. Among the substrate-related factors discussed are available surface area of cellulose which can be covered by lignin and thereby become unaccessible for cellulases [13], cellulose crystallinity, degree of polymerization and pore volume [14].

Native cellulose I exists in the form of fibrils, which are bundles of β-(1,4)-d-glucan polymer chains. Cellulose has a simple chemical structure but the arrangement of these chains into a supramolecular structure makes cellulose surprisingly complex. In the solid state, cellulose polymers are packed together and are described in terms of the spatial distribution of fibrils and fibril aggregates, the specific surface area (SSA), degree of crystallinity (DCr) and porosity. The supramolecular characteristics of these fibrils, such as lateral dimensions, are dependent on the species and isolation process [15]. The arrangement of the individual polymers in a fibril is highly dependent on the location within the fibril. Molecular dynamic simulations have shown that the cellulose structure at the surface is different from the crystalline bulk structure [16]. Analysis of cellulose-water interfaces has shown that the C4 atoms in cellulose chains located on top of different crystallographic planes have different mobilities [15].

**Figure 1:**
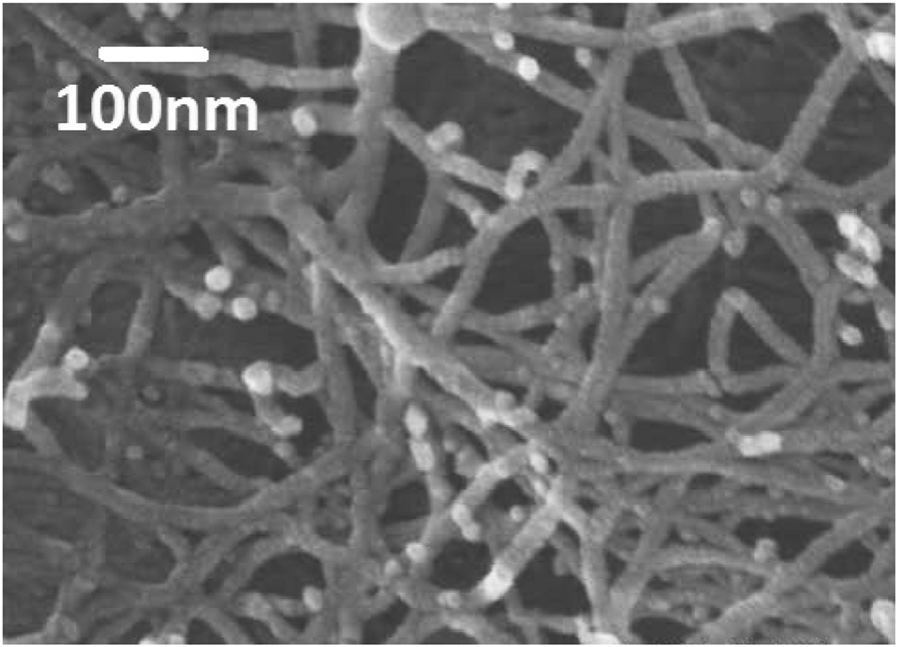
**A field emission scanning electron microscopy image of a deliginified kraft pulp fibre adapted from** [20] **(reprinted with modifications with permission)**. After removal of non-cellulose I material during the kraft cooking process, it has resulted in a highly porous fibre wall morphology where fibril aggregates form a network.

In most cases, cellulose fibrils are embedded in a lignocellulose matrix, where the cellulose content ranges from approximately 40 to 50% of the plant (dry weight) [4]. In case of cotton, cellulose is the major constituent (over 94%). Cellulose is also synthesized by bacteria and prokaryotes [17]. The cellulose I fibrils can assemble into larger supramolecular structures or fibril aggregates that constitute the cellulose network. During the isolation procedure of cellulose I-rich plant fibres, non-cellulose I components are removed and therefore, additional pores are formed in the fibre wall which create a porous cellulose network [18-20]. This network of cellulose has a complex orientation in space [20] (Figure 1).

Different fibril models have been proposed in the literature. In one of them, the fibril aggregates have a rectangular cross-section [21,22]. In the other, the fibril is composed of 36 elementary chains, in which it is assumed that the fibril aggregates have a hexagonal cross-section [23]. It has been shown that endo-β-1,4-glucanase and its isolated carbohydrate-binding domain adsorb preferentially on certain edges or faces of *Valonia* cellulose microcrystals [24]. It has been suggested that the faces preferred for the binding of enzymes have hydrophobic character [8,25,26]. These findings demonstrate the complexity of cellulose and the necessity to understand the mechanisms of cellulose hydrolysis in greater detail. The solubilized sugars released from cellulose are usually measured during the course of enzymatic hydrolysis, and the non-hydrolysed fraction is attributed to the recalcitrant nature of cellulose. This approach is very common in research on the production of bioethanol. In contrast, in research on forestry products, a great deal of attention is paid to the overall structure of cellulose fibres and the understanding of their reactivity in thermochemical processes [27].

Various imaging techniques have been used to investigate cellulose structure and cellulose-cellulase interactions, such as fluorescence microscopy, transmission electron microscopy, atomic force microscopy and scanning electron microscopy [10,28,29]. The drawback of these microscopic techniques is that only a very small part of the substrate can be visualized, from which conclusions are drawn regarding the whole substrate.

An interdisciplinary approach was adopted in the current study, combining knowledge from the fields of biotechnology, chemical pulping and cellulose structure, employing solid-state cross-polarization magic angle spinning carbon-13 nuclear magnetic resonance (CP/MAS ^13^C-NMR) with spectral fitting in order to characterize the supramolecular structure of cellulosic substrates during enzymatic hydrolysis. We investigated whether the supramolecular structure of cellulose, in terms of the spatial distribution of fibrils and fibril aggregates, the accessible surface area, crystallinity and porosity, can influence its hydrolysability. We believe that investigations of cellulose structures with nanometre resolution will provide a more precise picture of the process as both enzymes and the building blocks of cellulose have these dimensions.

## Results and discussion

### The model used for the supramolecular structure of cellulose

**Figure 2:**
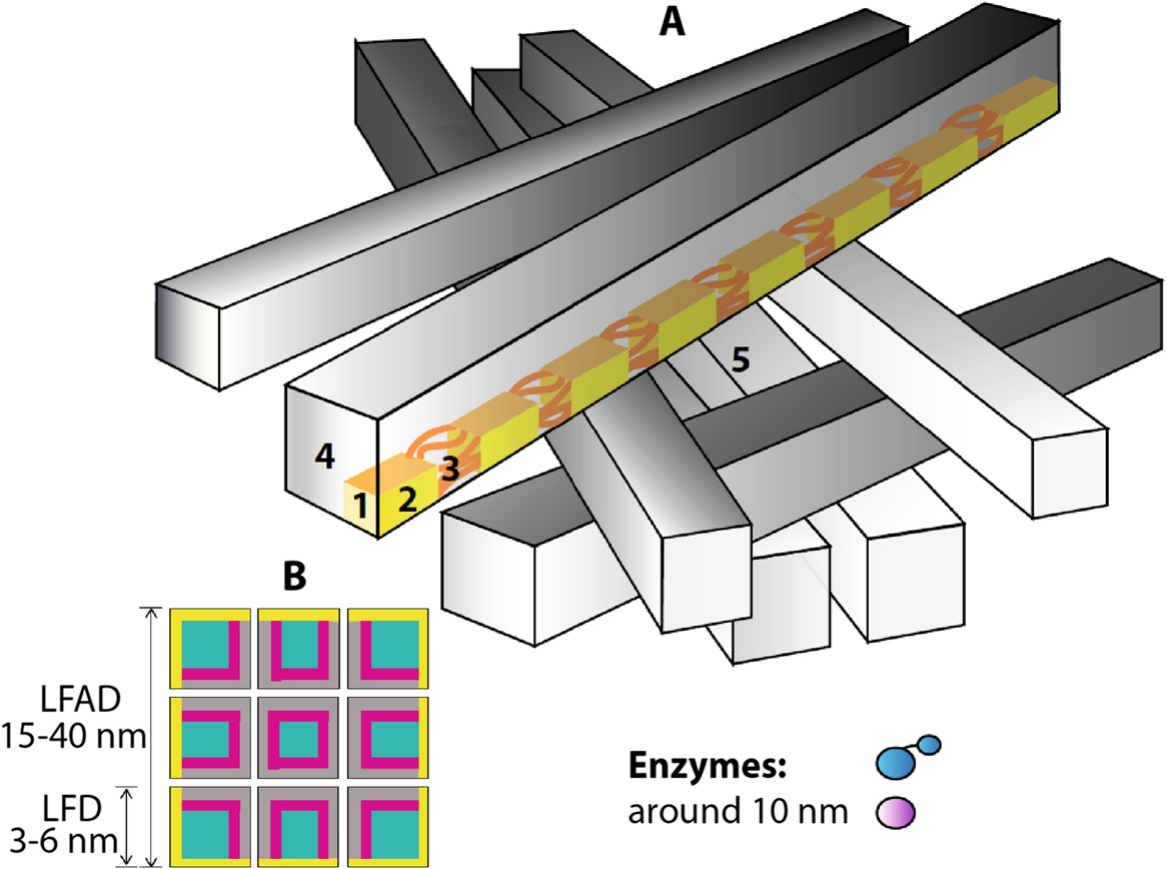
**Complexity of supramolecular structure of cellulose. A** Schematic representation of the model used in the present study to represent the supramolecular structure of cellulose, where fibril aggregates are shown as having square cross-sections with the following key elements: (1) fibril, a bundle of β-(1,4)-d-glucan polymers which is a mixture of the structures showing a high degree of three-dimensional order (crystalline) (2) and disordered (non-crystalline) (3) domains; (4) fibril aggregate, a structural element of cellulose composed of a bundle of fibrils; (5) pore, a cavity between fibril aggregates. **B** Front view of the model of the aggregated cellulose I fibrils used in calculations of lateral fibril aggregate dimensions (LFAD) and lateral fibril dimensions (LFD). The model differentiates between crystalline regions (turquoise), para-crystalline regions (magenta), accessible surface area (yellow) and inaccessible surface area that results from the close proximity of fibrils in a fibril aggregate (grey). Different enzymes are involved in the enzymatic hydrolysis of cellulose; some have a carbohydrate-binding module and a catalytic module, while others have only a catalytic module.

**Figure 3:**
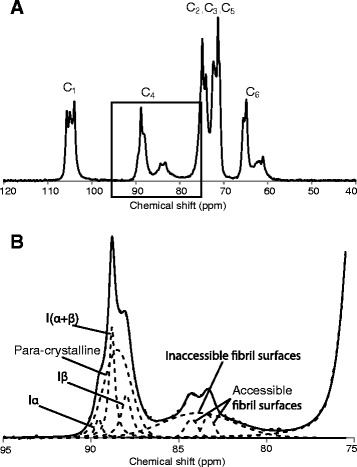
**A representative CP/MAS** ^**13**^ **C-NMR spectrum from cellulose, together with a typical spectral fitting result. A** CP/MAS ^13^C-NMR spectrum of cotton. **B** Enlargement of the C4 region of cotton cellulose. To determine the supramolecular structure of cellulose, the C4 region is fitted with a set of mathematical functions representing the signals originating from C4 carbons in cellulose Iα, cellulose I(α + β), cellulose Iβ, para-crystalline cellulose, C4 carbons in polymers residing at inaccessible fibril surfaces and C4 carbons in polymers residing at accessible fibril surfaces [27,31,32].

Collecting the knowledge from the literature, we built a model, emphasizing the complexity of supramolecular structure of cellulose, depicted in Figure 2. This model was used for interpreting our experimental data. Earlier results which have shown that during the isolation of the pulp fibres, i.e. removal of non-cellulosic material, fibril aggregates form a highly porous fibre wall morphology, were incorporated in the present model [20]. In our discussion of the results, we used the term fibre wall morphology which describes the network formed by the fibril aggregates inside the fibre wall remaining after the isolation procedure. Along the fibril axis, there are ordered (crystalline) and disordered (non-crystalline) regions [21,30,31]. However, it is not clear exactly how these regions are distributed within the fibril nor to which extent they occur. CP/MAS ^13^C-NMR measurements with spectral fitting [19,27,31,32] (Figure 2B) were done on the cellulosic substrates based on which the supramolecular structure of cellulose was represented in terms of the spatial distribution of fibrils and fibril aggregates, the accessible surface area, crystallinity and porosity. A representative CP/MAS ^13^C-NMR spectrum from cellulose, together with a typical spectral fitting result, is shown in Figure 3. It serves to emphasize that for efficient enzymatic hydrolysis, the surface of cellulosic substrates need to be accessible to the enzymes, implying that the typical fibre wall pore sizes need to be larger than the typical sizes of the enzyme molecules which are around 10 nm [26].

### Cellulosic substrates

Six cellulosic substrates with different supramolecular and physical properties were studied. Avicel PH-101 (Avicel) and cotton are commercially available celluloses. Both are non-lignin-containing materials and were delivered as dry materials. During the manufacture of Avicel, the cellulose pulp is subjected to acid hydrolysis and spray drying [33], resulting in a powder containing particles with remnants of the fibre wall morphology. Avicel is used extensively in enzymatic studies as a model substrate for cellulose. Cotton is a fibrous material with essentially intact fibre wall morphology. In an attempt to increase the SSA of cotton, it was treated with sodium hydroxide (cotton + NaOH), under conditions not leading to any significant conversion of cellulose I to cellulose II nor changes in chemical composition. Never-dried, oxygen-delignified, softwood pulp from pre-hydrolysis soda cooking technique (OPHS-ND) was used to represent cellulose isolated from wood. The OPHS-ND substrate can in a sense represent the best possible case of commercial pulps specifically designed for high enzymatic reactivity. This was achieved by combining high cellulose content with a suitable balance between fibre wall SSA and fibre wall average pore size. In order to reduce the possible influence of fibre wall morphology on enzymatic reactivity, cellulose nano-particles, sometimes referred to as nano-crystalline cellulose (NCC), were manufactured from OPHS-ND by prolonged hydrochloric acid hydrolysis at elevated temperature (NCC-OPHS-ND). The result was a colloidal suspension of cellulose nano-particles resulting from the removal of the fibre wall morphology. Part of the OPHS-ND material was also subjected to drying and rewetting (OPHS-D). This is known to induce changes in the supramolecular structure of cellulose I, partly responsible for the phenomenon known as hornification, traditionally measured as a reduction in the water-binding capacity of pulp [27]. It should be noted that drying induces little or no change in the chemical composition [34]. The chemical compositions of the substrates, representing wood [35] and cotton, are given in Table 1. It should be emphasized that all the substrates used in this study were cellulose-rich, all having cellulose content typically above 96%, and the main differences between the substrates were of a structural nature.

**Table 1:** **Chemical composition (in percentage) of the substrates studied**

**Substrate**	**Avicel**	**OPHS-ND and OPHS-D**	**NCC-OPHS-ND**	**Cotton and cotton + NaOH** ^**a**^
Acid-insoluble lignin	n.a.	0.5	n.d.	n.a.
Acid-soluble lignin	n.a.	0.5	n.d.	n.a.
Extractives	n.d.	<0.25	n.d.	0.7
Ash content	n.d.	<0.1	n.d.	0.2
Xylose	2.2	1.5	0.8	0.5
Mannose	0.9	0.8	0.8	0.1
Arabinose	<0.1	<0.1	<0.1	0.1
Galactose	<0.1	<0.1	<0.1	0.2
Glucose	96.8	96.7	98.4	98.2

Major hemicelluloses in softwood (OPHS-ND, OPHS-D and NCC-OPHS-ND substrates) are (galacto)glucomannan, galactoglucomannan and arabinoglucuronoxylan and major hemicelluloses in hardwood are glucuronoxylan and glucomannan [35]. ^a^Carbohydrate composition analysis was done on cotton substrate. Since cotton has such a high degree of purity, NaOH treatment is not expected to change its chemical composition. n.a., not applicable; n.d., not determined.

### Initial cellulose supramolecular structure and the hydrolysability of the cellulosic substrates

The enzymatic hydrolysability, measured as the conversion yield of cellulosic substrates to glucose after 2 days, was quite different for the six substrates investigated (Table 2). Although the experimental conditions were identical, the conversion yield of the cellulosic substrates ranged from 2% to 91%. This was interpreted as an indication that the structural properties of cellulose may affect enzymatic hydrolysability. NMR results given in Table 2 provide average values of the supramolecular properties of the sample before enzymatic hydrolysis and after 2 days of hydrolysis. For example, after 2 days of hydrolysis, 31% of the cellulose in Avicel was hydrolysed which means that 69% residue remained, on which a supramolecular structure was measured.

**Table 2:** **Structural characteristics of the substrates before and after 2 days of enzymatic hydrolysis**

**Substrate**	**Physical state**	**Size**	**Before hydrolysis**				**After 2 days of hydrolysis**				
			**LFD (nm)**	**LFAD (nm)**	**SSA (m** ^**2**^ **g** ^**−1**^ **)**	**DCr (%)**	**LFD (nm)**	**LFAD (nm)**	**SSA (m** ^**2**^ **g** ^**−1**^ **)**	**DCr (%)**	**Conversion yield (%)**
Avicel	Dried powder	Micro (round particles)	4.5 ± 0.1	23.7 ± 1.1	113 ± 5	56 ± 3	4.5 ± 0.1	20.4 ± 0.6	131 ± 4	56 ± 1	30.5 ± 0.2
Cotton	Dried fibres	Milli (in length)	5.8 ± 0.1	29.7 ± 1.2	90 ± 4	65 ± 2	n.d.	n.d.	n.d.	n.d.	2.1 ± 0.4
Cotton + NaOH	Dried fibres	Milli (in length)	6.1 ± 0.1	26.2 ± 0.9	102 ± 4	66 ± 2	n.d.	n.d.	n.d.	n.d.	6.3 ± 1.1
OPHS-ND	Never-dried fibres	Milli (in length)	4.7 ± 0.1	17.5 ± 0.8	153 ± 7	57 ± 1	5.8 ± 0.2	20.1 ± 1.1	132 ± 7	65 ± 3	67.7 ± 0.2
OPHS-D	Dried fibres	Milli (in length)	4.7 ± 0.1	28.3 ± 0.7	94 ± 2	57 ± 1	5.0 ± 0.1	29.5 ± 1.1	90 ± 3	59 ± 2	46.2 ± 5.2
NCC-OPHS-ND	Never-dried particles	Nano (rod-like particles)	5.6 ± 0.2	28.9 ± 1.7	92 ± 5	63 ± 2	5.5 ± 0.2	20.6 ± 1.1	130 ± 7	63 ± 3	90.7 ± 2.1

LFD, lateral fibril dimensions (or fibril thickness); LFAD, lateral fibril aggregate dimensions (or aggregate thickness); SSA, specific surface area; DCr, degree of crystallinity estimated from CP/MAS ^13^C-NMR measurements; n.d., not determined.

Only weak correlations were found between initial SSA and enzymatic conversion yield of the cellulose substrates. No clear correlations were seen between the DCr before hydrolysis and conversion yield. The sample with the highest conversion yield (NCC-OPHS-ND) had one of the highest DCr before hydrolysis.

In the model for supramolecular cellulose I used here (Figure 2), the SSA accessible to enzymes is assumed to be the envelope area of cellulose fibril aggregates computed from the LFAD, and the lateral dimensions of the individual fibrils are therefore assumed to be of less importance. Ultimately, the fibril aggregate envelope surface is composed of fibril surfaces, but aggregation prevents access to the whole fibril surface area within a fibril aggregate. It is therefore assumed that the fibril aggregate envelope surface constitutes an upper limit of the SSA accessible to the enzymes during the initial stage of hydrolysis. This is consistent with the data in Table 2, since no strong correlations were found between the LFD of the starting materials and the ability of the enzymes to hydrolyse them.

Furthermore, in the cellulose model used here (Figure 2), it is assumed that there is a relation between the DCr and LFD. Since the largest structural element of cellulose capable of maintaining crystalline cellulose I lattice is the cellulose fibrils, the DCr is ultimately limited by the LFD. The CP/MAS ^13^C-NMR spectrum recorded on cellulose I shows separate signals originating from C4 carbons in polymers residing on fibril surfaces and C4 carbons in polymers residing in the fibril interior (Figure 3). The difference in carbon-13 chemical shift is in the range of 4 to 6 ppm, indicating a significant difference in polymer conformation between surface and interior polymers. This is further supported by previous measurements of laboratory frame carbon-13 spin–lattice relaxation times [15,32]. The C4 carbons on the fibril surfaces show a significantly shorter carbon-13 spin–lattice relaxation time than any other structural domain detected, and the difference in relaxation time can be ten or more times that for the interior structures. This means that the polymers on fibril surfaces are quite mobile compared to interior polymers. For these reasons, it is assumed in the NMR model that surface polymers, on both accessible and inaccessible surfaces, are not identical to polymers in the cellulose I crystal lattice and that they therefore do not contribute to the DCr. This might have two consequences related to the cellulose structure. Firstly, even in the hypothetical case of perfect longitudinal polymer ordering in a fibril, the small lateral dimensions of a cellulose I fibril prevent the occurrence of completely crystalline cellulose I. Secondly, since the polymers at the fibril surface are not considered to be in a crystalline state, enzymes attach to fibril surfaces where the polymers are in a conformational and dynamical state quite distinct from that of the polymers in the crystalline fibril interior. This hypothesis is the basis of our suggested cellulose model, and it is supported by the lack of any strong correlations between the LFD or the DCr, which is inversely proportional to LFD and the conversion yields (Table 2).

In a previous study by our group, enzyme production during the filamentous fungus *T. reesei* growth on cellulose-rich pulps isolated from softwood and NCC-OPHS-ND was compared with growth of the fungus on Avicel. It was found that enzyme production by the filamentous fungus *T. reesei* showed the highest protein production when Avicel was used as a carbon and energy source for the fungus and that protein production levels were significantly lower when cellulose-rich pulps or NCC-OPHS-ND were used as carbon sources [36]. This observation led us to hypothesize that there may exist a relation between the structural properties of the substrate used for the fungal growth and the capacity of the produced enzymes to hydrolyse the same substrate. In the present study, a commercial mixture of cellulases from *T. reesei* and excess of β-glucosidase to prevent cellobiose inhibition were used. Interestingly, in the present study, Avicel showed a lower conversion yield than the cellulose-rich pulp samples. This is opposite to the trends observed for protein production among our samples and suggests that cellulosic substrates that are more recalcitrant to enzymatic hydrolysis may result in higher enzyme production when they are used as carbon and energy sources for fungi.

### Changes in the supramolecular structure of cellulose resulting from enzymatic hydrolysis

The characteristics of the Avicel, OPHS-ND, OPHS-D and NCC-OPHS-ND substrates were measured before and after enzymatic hydrolysis. No significant change in LFD was observed for Avicel or NCC-OPHS-ND as a result of hydrolysis. This could be because these substrates had been subjected to acid hydrolysis prior to enzymatic hydrolysis.

The LFD for the OPHS-ND and OPHS-D substrates increased as a result of enzymatic hydrolysis, significantly in the case of OPHS-ND and slightly in the case of OPHS-D. Interestingly, the observed increase in LFD for OPHS-ND after enzymatic hydrolysis (from 4.7 to 5.8 nm) was about the same as the increase in LFD observed between OPHS-ND and the NCC-OPHS-ND (from 4.7 to 5.6 nm) as the result of acid hydrolysis of OPHS-ND during the manufacturing of NCC-OPHS-ND. It appears that the observed increase in LFD in this case is independent of whether acid or enzymatic hydrolysis is used. The mathematical model used estimates the DCr based on the LFD. Therefore, the observed increase in the DCr as the result of enzymatic hydrolysis observed for OPHS-ND, OPHS-D and NCC-OPHS-ND is simply a mathematical reflection of the observed increase in LFD.

If the longitudinal structure of cellulose I fibrils is as illustrated in Figure 2, with recurring regions of non-crystalline polymers along the fibril axis, preferential removal of these zones could result in an apparent increase in LFD. The calculations of LFD and LFAD are based on ratios of relative signal intensities obtained by spectral fitting of the NMR data. However, the mathematical model does not accommodate longitudinally recurring non-crystalline regions, and if these are in fact present, they will constitute a source of error in the estimates of LFD and LFAD. The signal intensity affected by the presence of a signal intensity from such non-crystalline regions will be that from the C4 atoms in polymers present on the surface of inaccessible fibrils, i.e. the broad signal centred at about 83 to 84 ppm (Figure 3). If the longitudinally recurring non-crystalline zones are preferentially attacked by hydrolysis without any other significant structural effects, the mathematical model will predict an increase in LFD accompanied by a (small) decrease in LFAD. No such observations were made for any of the substrates in this study (Table 2), as the result of acid hydrolysis or enzymatic hydrolysis. Although the treatment conditions used elsewhere are harsher, both NMR and wide-angle X-ray scattering method (WAXS) have shown the same trends, increasing LFD/crystallite size as a result of treatment [37]. As shown by both, NMR and X-ray, the increase in LFD is not a measurement artefact. Something does happen with the LFD as the result of acid and enzymatic hydrolysis, though we cannot explain the mechanism. We therefore conclude that longitudinally recurring regions of non-crystalline polymers may be present along the axis of the fibril, which may be more susceptible to hydrolysis, but the amount of material in these regions constitutes only a small fraction of the fibril. Therefore, another mechanism, or mechanisms, must be responsible for the observed increase in LFD resulting from hydrolysis.

Interestingly, the substrate showing the highest DCr before hydrolysis was the NCC-OPHS-ND sample, which showed no change in LFD (or DCr) after enzymatic hydrolysis, but still showed the highest conversion yield.

The two samples exhibiting the greatest changes in LFAD during enzymatic hydrolysis were Avicel and NCC-OPHS-ND. The mechanism behind these observations is not known, but both substrates had been subjected to acid hydrolysis prior to enzymatic hydrolysis. The structural changes induced by acid hydrolysis may make it easier for the enzymes to split the fibril aggregates apart during enzymatic hydrolysis, possibly due to fibril or fibril aggregate shortening which might occur during the acid hydrolysis step. Furthermore, it can be that the lack of change in LFD following enzymatic hydrolysis in the substrates having been subjected to acid hydrolysis could indicate that no significant lateral erosion took place during enzymatic hydrolysis. It is also worth noting that the sample showing the greatest decrease in LFAD as the result of enzymatic hydrolysis was NCC-OPHS-ND (29%), which was never dried, while the decrease in LFAD for Avicel, which was dried, was less (14%).

In the mathematical model used to interpret the NMR data, LFAD is assumed to be inversely proportional to SSA. In contrast to LFD, LFAD is known to vary depending on the isolation procedure used [18]. The OPHS-D substrate was made by drying OPHS-ND. During the drying of the OPHS pulp, there was an irreversible increase in LFAD (Table 2). This could be explained as an irreversible increase in the degree of aggregation with an associated decrease in SSA [18,27].

### The effect of enzymatic hydrolysis on degree of crystallinity

One common explanation of the slowing down or cessation of the enzymatic hydrolysis of cellulose is that after the more ‘easily accessible’ cellulose for enzymes has been converted to sugars, the remaining more ‘difficult’, i.e. crystalline, cellulose is recalcitrant to enzymatic hydrolysis [30]. However, spectral fitting of the CP/MAS ^13^C-NMR spectra revealed no direct correlations between the DCr before enzymatic hydrolysis and the conversion yield. Furthermore, in some substrates the DCr did not change as a result of enzymatic hydrolysis. In the model of the cellulose supramolecular structure, most cellulose fibrils are part of aggregates, which means that a high proportion of the fibril surface area and crystalline regions are in the interior of the aggregates (Figure 2), preventing direct enzymatic attack during enzymatic hydrolysis, at least during the initial stages of hydrolysis. It has been observed in recent studies that there were no changes in the DCr of Avicel during the course of hydrolysis [9,38], in agreement with our findings. It has been proposed that the DCr remains constant during enzymatic hydrolysis due to the simultaneous hydrolysis of crystalline and amorphous domains [39].

The NCC-OPHS-ND substrate was made by acid hydrolysis of the OPHS-ND pulp using concentrated (2.5 M) hydrochloric acid at elevated temperature (95°C to 100°C) for 17 h. The NCC-OPHS-ND sample was the material remaining after acid hydrolysis. Traditionally, it is assumed that the so-called amorphous domains in cellulose are removed during acid hydrolysis. For this reason, the explanation according to Puls and Wood [39] seems less likely for explaining the unchanging DCr observed during the subsequent enzymatic hydrolysis of the NCC-OPS-ND sample since little or no ‘amorphous’ cellulose remained in the starting material for the enzymatic hydrolysis. This may also be the case with Avicel since acid hydrolysis is employed during its manufacture [33].

No change in crystallinity was observed for Avicel, OPHS-D or NCC-OPHS-ND substrates after 2 days of enzymatic hydrolysis. The OPHS-ND substrate showed a significant increase in the DCr after enzymatic hydrolysis but, as discussed above, without any accompanying decrease in LFAD (Table 2). Based on the model of cellulose I as a supramolecular structure and the findings presented in Table 2, we conclude that the degree of cellulose I crystallinity is not a major factor controlling enzymatic hydrolysability.

### Hydrolysability is improved when the pore size of the substrate is larger than the size of the enzyme molecules

The efficiency of the enzymatic hydrolysis of cellulose depends on the surface area accessible to the enzymes. An important parameter affecting this is the pore size of the material in relation to the molecular size of the enzymes [14]. The projection of a bound cellobiohydrolase I molecule onto a cellulose surface has been estimated to be around 10 nm [26]. In the present work, we combined SSA measurements obtained from CP/MAS ^13^C-NMR spectra with results from fibre saturation point (FSP) measurements to estimate the average pore sizes in water-swollen samples. It should be noted that the FSP method used is not applicable to substrates lacking fibre cell wall morphology, such as the NCC-OPHS-ND substrate.

In cellulose I-rich substrates with remaining fibre wall morphology, a pore system exists in a water-swollen state of the samples. Fibre wall pore sizes are in the range of 1 to 100 nm [40], covering the range of sizes of typical enzyme molecules. Pore size could thus influence the penetration of enzymes into porous fibre walls and their subsequent attack on the cellulose surfaces available inside the water-swollen fibre wall. Measuring pore size distributions, or average pore sizes, in a water-swollen fibre wall or a fragment of such a wall is quite complicated and prone to errors, partly due to the soft nature of the material when in a water-swollen state. Stone and Scallan have developed a method based on solute exclusion that can be used to estimate the total water content of the fibre wall pore system when in a water-swollen state, which they call the FSP [41]. When the results of FSP measurements are combined with NMR estimates of the SSA, also measured when the sample is in a water-swollen state, the average pore size of the fibre wall can be estimated [19]. One advantage of combining FSP and NMR results is that estimates of the average pore size can be obtained without the need for sample drying, which often causes structural changes. Larsson et al. have shown that for cellulose-rich samples, the often employed nitrogen- or krypton-adsorption Brunauer-Emmett-Teller (BET) method [42] underestimates the SSA by as much as a factor of two, resulting in a similar overestimation of the pore size by a factor of two [19]. Köhnke et al. have recently published estimates of FSP and SSA for Avicel PH-105, the latter using nitrogen BET [43], and it can be expected that values of the average pore size based on these measurements will be overestimated by a factor of about two, compared with those made using the method described by Larsson et al. [19].

During enzymatic hydrolysis, the NCC-OPHS-ND sample is a sol (solid particles in a liquid) without any well-defined pore size. In order to assess if the cellulose concentration used could result in a cellulose nano-particle density so high that it might exert restrictions on the accessibility of the enzyme to the cellulose particles, an order-of-magnitude estimate of the average inter-particle distance in the reaction medium was made (See Additional file 1). The estimate showed that at the cellulose nano-particle concentration used, the average inter-particle distance in a well-dispersed system, during sufficient agitation, was an order of magnitude larger than the typical enzyme molecules. The interpretation was that no significant restrictions in accessibility of enzymes to cellulose particles were imposed by the cellulose nano-particle density in the reaction medium. A possible limiting factor for enzymatic conversion in this system could be the amount of cellulose nano-particle SSA.

**Figure 4:**
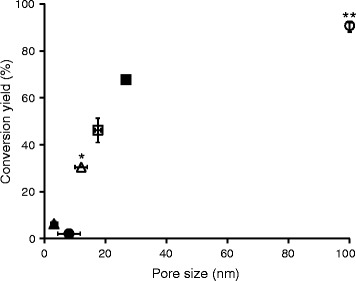
**Average pore size in the substrates before enzymatic hydrolysis, together with conversion yields after 2 days of enzymatic hydrolysis: cotton + NaOH (black triangle), cotton (black circle), Avicel (white triangle), OPHS-D (white square), OPHS-ND (black square) and NCC-OPHS-ND (white circle).** Errors in the conversion yields (%) are represented by the minimum and maximum values of two replicates. *The value for the average pore size in Avicel was calculated from the data given by Köhnke et al. 20 (±1) nm (FSP = 0.47 ± 0.02 g g^−1^, SSA(BET) = 47 ± 1 m^2^ g^−1^) [43]. Taking into account the underestimation of the SSA by the BET method, the estimated pore size of Avicel is probably in the range 10 to 14 nm, calculated according to Larsson et al. [19]. ** Average pore size is >100 nm (see Additional file 1).

Investigating the relationship between the average pore size and the hydrolysability of the substrates showed that conversion yield increased with increasing pore size of the substrate (Figure 4). A significant reduction in conversion yield was observed when the average pore size was in the order of, or smaller than, the size of typical enzyme molecules. Pore size is affected by drying conditions. It has been shown that LFAD are related to the rate at which water is removed from the cellulose fibres during drying. Pore size also depends on the presence of spacers. Spatial distribution of spacers such as hemicellulose and lignin can limit the degree of irreversibility of the structural changes caused by drying [18,27,44]. The OPHS-D substrate was prepared by drying OPHS-ND substrate, and the average pore size decreased from about 27 to 18 nm, but was still larger than the enzyme molecules (Figure 4). These results suggest that the average pore size may be an important factor governing the enzymatic hydrolysability of the substrates.

The NCC-OPHS-ND and OPHS-D substrates were made from the same material, OPHS-ND, and thus had similar SSA before hydrolysis, 92 and 94 m^2^ g^−1^ (Table 2), respectively, which suggests that the observed difference in conversion between these two substrates is dominated by effects related to pore size/enzyme accessibility.

We investigated whether there were any correlations between substrate characteristics before enzymatic hydrolysis and the conversion yield measured after hydrolysis. During hydrolysis, the action of the enzymes may alter both the SSA and average pore size. Since identical reaction conditions were used throughout the experiments, the effects on conversion yield will be cumulative, making kinetic factors during the early stages of hydrolysis important for the conversion yield.

It should be noted that average pore sizes were used in this study. Pore size distributions and the detailed structural characteristics of the pore system, such as bottlenecks and dead-end pores may considerably reduce the SSA accessible to the enzymes. This means that the correlation between average pore size and conversion yield may not be perfect but, as the data in Figure 4 suggests, the impact of such pore system characteristics on the conversion yield is probably most pronounced in substrates where the average pore size is in the order of, or smaller than, the typical size of enzymes molecules.

It is also interesting to note that the substrate resulting in the highest conversion yield was NCC-OPHS-ND, which was a sol of cellulose nano-particles without any well-defined pore size, exhibiting a 91% conversion yield and the highest DCr. Its effective pore size during hydrolysis is the average inter-particle distance in the suspension, which is significantly larger than any enzyme molecule. Prolonged sedimentation or centrifugation gives an apparently dense pellet of cellulose nano-particles, but little is known about the average pore size in such pellets. When carrying out the enzymatic hydrolysis experiments, agitation was performed to prevent the formation of dense non-porous regions of nano-particles in the reaction tube. It was thus assumed that during enzymatic hydrolysis of NCC-OPHS-ND substrate the average distance between cellulose nano-particles in the reaction tube was much greater than the typical dimensions of enzyme molecules, imposing no significant additional hindrance on the enzyme molecules.

### Possible mechanisms in enzymatic hydrolysis of cellulose

Several aspects of the supramolecular structure of cellulose may be affected during enzymatic hydrolysis, such as scission of fibrils and fibril aggregates, preferential hydrolysis of non-crystalline zones along the fibril axis, lateral erosion and cleavage of liberated fibril aggregates into individual fibrils. The interpretation of the data in Table 2 is, to a large extent, based on CP/MAS ^13^C-NMR analysis. It would be useful to evaluate the changes in NMR spectra resulting from changes in the supramolecular structure of cellulose that may occur during the time course of enzymatic hydrolysis. Since NMR spectral features mainly results from the lateral properties of the supramolecular cellulose I structure, little can be concluded about changes in the longitudinal properties. It is not until the LFD becomes so small that the signal from reducing end groups appears at about 96 ppm that any longitudinal characteristics affect spectra. The signal-to-noise ratio of the NMR spectra is typically such that the signal from reducing end groups becomes detectable when the degree of polymerization is below 150 to 200 anhydroglucose units. Theoretically, lateral erosion should be easily detected in these spectra. Successive erosion of the fibrils comprising the fibril aggregate surface is expected to be detected as a decrease in the average LFD. Similarly, the cleavage of fibril aggregates into separate fibrils is expected to result in a decrease in LFAD, possibly accompanied by a decrease in LFD, as some of the liberated fibrils start to become laterally eroded. When there are significant amounts of non-crystalline material along the fibril axis, preferential hydrolysis of these regions would lead to an apparent increase in LFD (and a corresponding increase in the DCr), accompanied by a slight decrease in LFAD, as discussed above. Other mechanisms may also alter the supramolecular structure of cellulose I. Several mechanisms may be working simultaneously, enhancing or counteracting the expected spectral changes, making it difficult to relate the observed spectral changes to specific mechanisms. Despite the complexity of the situation, a few cases in Table 2 stand out, and these will be discussed below.

The increase in LFD (or DCr) observed as the result of acid hydrolysis of the OPHS-ND substrate (NCC-OPHS-ND) and as the result of enzymatic hydrolysis of the OPHS-ND substrate was significant, about 20% in both cases, without any significant decrease in the LFAD. The magnitude of the change in LFD, together with the lack of decrease in LFAD, indicates that the hydrolysis of longitudinally distributed non-crystalline regions is not a likely explanation. Also, the less pronounced increase in LFD resulting from enzymatic hydrolysis of the OPHS-D substrate is difficult to explain, unless drying and rewetting would decrease the extent of longitudinally distributed non-crystalline regions. If the removal of longitudinally distributed non-crystalline regions is the explanation for the increase in LFD observed during hydrolysis, this would suggest that the actual fibril width of isolated wood-based cellulose I is 5 to 6 nm, rather than the 3 to 4 nm normally quoted. It is therefore likely that some other mechanism is responsible for the observed increase in LFD.

Enzymatic hydrolysis of NCC-OPHS-ND did not result in any change in the LFD or DCr, while the LFAD changed from 29 to 21 nm due to the hydrolysis of about 91% of the material. It is difficult to explain these observations by any other mechanism than successive shortening of the structural elements during enzymatic hydrolysis. It is also plausible that as the fibril aggregates become shorter, the cohesion between individual fibrils may became weaker, resulting in a decrease in LFAD due to cleavage of the fibril aggregates.

## Conclusions

In the present study, we used a set of substrates with high cellulose content to investigate if the supramolecular structure of cellulose could influence the enzymatic hydrolysability. Initial cellulose supramolecular properties were used to interpret conversion yield measured after 2 days of enzymatic hydrolysis. Larger pore sizes in the cellulosic substrates resulted in higher conversion yield. We found no pronounced correlations between conversion yield and the SSA and LFD. Both acid and enzymatic hydrolysis can increase the LFD, but no plausible mechanism leading to the observed increase in LFD could be given. The substrate with the highest initial DCr, NCC-OPHS-ND, showed the highest conversion yield (91%), without any accompanying change in LFD, seemingly in conflict with a mechanism based on lateral erosion.

## Methods

### Substrates

Six different cellulosic substrates were used in this study (Table 3). Avicel® PH-101 (Fluka BioChemika, Cork, Ireland) has been used in many studies and therefore served as a reference. Two cotton (Original Topz®, Gunry AB, Kungsbacka, Sweden) substrates were used: one as delivered and in other case 1% of cotton (*w*/*v*) was treated with 5% NaOH (*w*/*v*) for 1 h at room temperature without stirring, in an attempt to increase the SSA. After treatment with NaOH, the cotton was washed with deionized water, until the pH was the same as that of the washing water. The sample material was filtered through a Miracloth (Calbiochem, La Jolla, CA, USA) and stored at 4°C in airtight plastic bags until it was used in enzymatic hydrolysis experiments. OPHS was produced from softwood biomass consisting of an industrially chipped and screened mixture of 40% Scots pine (*Pinus sylvestris*) and 60% Norway spruce (*Picea abies*). After screening, chips with bark and knots were removed by hand from the accept fraction, chip thickness 2 to 8 mm. The chips were subjected to pre-hydrolysis followed by an alkaline soda cooking according to the procedure described elsewhere [36,45]. The chemical composition of the softwood biomass was 28.3% acid-insoluble (Klason) lignin, 0.3% acid-soluble lignin, 41.1% cellulose, 9.5% xylan, 17.7% (galacto)glucomannan, 2.8% extractives and 0.3% ash. Never-dried (ND) and dried (D) OPHS substrates were used in the study. To produce OPHS-D substrate, OPHS-ND was gently stirred in deionized water until it became homogeneous. It was then filtered through a Miracloth, and the solid material was manually separated into small pieces, which were left at room temperature for 2 h and then dried overnight in an oven at 85°C. This material was stirred in deionized water until the mixture became homogeneous, filtered through a Miracloth and stored at 4°C in airtight plastic bags until it was used in the enzymatic hydrolysis experiments. NCC-OPHS-ND substrate was produced from OPHS-ND according to the procedure described elsewhere and stored in excess of deionized water in a bottle with a lid in room temperature [36].

**Table 3:** **Description of the substrates used in this study**

**Abbreviation**	**Full name**	**Treatment**	**Origin**
Avicel® PH-101	Commercial microcrystalline cellulose	Acid hydrolysis of wood pulp	Purchased
Cotton	Pharmaceutical grade cellulose	None	Purchased
Cotton + NaOH		NaOH treatment of cotton	This study
OPHS-ND	Oxygen-delignified pre-hydrolysis soda pulp-never-dried	Pre-hydrolysis soda cooking and oxygen delignification of prehydrolysis soda pulp from softwood	This study
OPHS-D	Oxygen-delignified pre-hydrolysis soda pulp-dried	Drying of OPHS-ND pulp	This study
NCC-OPHS-ND	Nano-crystalline cellulose	Acid hydrolysis of OPHS-ND pulp	This study

### Analysis of the chemical composition of the substrates

The ash content of biomass and OPHS was determined according to ISO 1762. The biomass and OPHS were extracted by acetone prior to analysis of the carbohydrates and lignin to determine the amount of acetone-extractable matter (extractives), according to SCAN-CM 49:03 [46]. To analyse the carbohydrates, samples were hydrolysed at 121°C in an autoclave with 0.4 M H_2_SO_4_, according to SCAN-CM 71:09 [47]. The solubilized monosaccharides were quantified using high-performance anion exchange chromatography with a Dionex ISC-5000 system coupled to a CarboPac PA1 (250 mm × 4 mm i.d.) column (Dionex, Stockholm, Sweden) and a pulsed amperometric detector (HPAEC-PAD). To determine the lignin content, the samples were hydrolysed with sulphuric acid and then filtered, and the acid-insoluble lignin (Klason lignin) was determined gravimetrically according to TAPPI T 222 om-02 [48]. The acid-soluble lignin was measured by UV spectrophotometry at 205 nm, according to TAPPI UM 250 [49]. MilliQ water was used as a blank and for dilution of the hydrolysate. The acid-soluble fraction was calculated using an absorption coefficient of 110 L g^−1^ cm^−1^. The total content of lignin was assumed to be the sum of the acid-soluble and acid-insoluble fractions. Samples were analysed in duplicates.

### Characterization of the substrates by CP/MAS ^13^C-NMR

All samples were wetted with deionized water in the range of 40 to 60% water content and packed uniformly in a zirconium oxide rotor. Recording spectra from wet rather than dry samples give a higher apparent resolution. The CP/MAS ^13^C-NMR spectra were recorded in a Bruker Avance III AQS 400 SB instrument operating at 9.4 T. All measurements were carried out at 295 (±1) K with a magic angle spinning (MAS) rate of 10 kHz. A 4-mm double air-bearing probe was used. Data acquisition was performed using a cross-polarization (CP) pulse sequence, i.e. a 2.95 μs proton 90 degree pulse and an 800-μs ramped (100 to 50%) falling contact pulse, with a 2.5 s delay between repetitions. A SPINAL64 pulse sequence was used for 1H decoupling. The Hartmann-Hahn matching procedure was based on glycine. The chemical shift scale was calibrated to tetramethylsilane (TMS ((CH_3_)_4_Si)) scale by assigning the data point of maximum intensity in the alpha-glycine carbonyl signal to a shift of 176.03 ppm. A total of 4,096 transients were recorded from each sample, leading to an acquisition time of approximately 3 h. The software for spectral fitting was developed at Innventia AB and is based on a Levenberg-Marquardt algorithm [31]. All computations were based on integrated signal intensities obtained from spectral fitting [32]. To determine the supramolecular structure of cellulose, the C4 region is fitted with a set of mathematical functions representing the C4 signal intensity originating from C4 carbons in cellulose Iα, cellulose I(α + β), cellulose Iβ, para-crystalline cellulose, C4 carbons in polymers residing on inaccessible fibril surfaces and C4 carbons in polymers residing on accessible fibril surfaces. The integrated signal intensities obtained from the spectral fitting procedure are subsequently used for the calculation of the supramolecular characteristics of cellulose I, based on a cellulose model assuming square cross-sections of both fibrils and fibril aggregates [31]. More specifically, the LFAD, LFD, SSA and DCr were determined. The SSA for cellulose I was calculated from LFAD by assigning a density of 1,500 kg m^−3^ to cellulose I [27]. The errors given for LFD, LFAD, SSA and DCr are the standard error of the mean with respect to the quality of the fit. After convergence of the fitting procedure (using a chi^2^ objective function with respect to spectral noise) the parameter standard errors (asymptotic standard errors) are computed as the square root of the sum squared residuals multiplied by the corresponding diagonal element from the variance-covariance matrix, divided by the number of degrees of freedom. The number of degrees of freedom is set to the number of data points reduced by the number of fitted parameters. The variance-covariance matrix is the inverse of the transpose Jacobian matrix multiplied with the Jacobian matrix using unit weights. The Jacobian matrix is estimated from analytical partial parameter derivatives of all adjusted parameters calculated at each spectral point. The aim of this procedure is to estimate an uncertainty in the parameter mean value, not to establish a measure representative of the variability of an underlying population of fibril aggregates widths.

### Fibre saturation point measurements

FSP measurements were conducted according to Stone and Scallan [41]. Water-swollen sample material with a known solid content was mixed with a dextran solution of known concentration (approximately 1%, dextran mass/solution mass) (CAS No. 9004-54-0, Dextran 2000, from Leuconostoc spp., molecular mass approx. 2,000 kDa; Sigma-Aldrich, Stockholm, Sweden) in deionized water, approximately 1 mass unit of wet sample mass being mixed with 3 mass units of dextran solution. After mixing, the sample was stored in a sealed vessel at room temperature for 3 days to equilibrate. A liquid sample was subsequently taken and filtered through a Puradisc syringe filter (Whatman, Maidstone, UK) equipped with a 0.45-μm polytetrafluoroethylene (PTFE) membrane in a polypropylene housing (VWR International AB, Stockholm, Sweden). The concentration of dextran in the sample was determined using a calibration curve established for the optical rotation of polarized light measured using a Polartronic NH8 polarimeter (Schmidt + Haensch, Berlin, Germany) operating at 589 nm, with a resolution of 0.005°. The calibration curve was computed using three dextran concentrations: approximately 0.5%, 1.0% and 1.5% (dextran mass/solution mass), covering the range of all measurements. Dynamic light scattering was used to determine the hydrodynamic diameter of the dextran molecules at high dilution in deionized water (Zetasizer ZEN3600; Malvern Instruments Ltd., Malvern, UK), using a He-Ne 4.0 mW, 633 nm laser and a detector angle of 178°. The hydrodynamic diameter was found to be 101 ± 2 nm with a polydispersity index of 0.2, measured at a dextran concentration of 0.15 g dextran per litre solution. Based on the determined size of the dextran, the results obtained for the FSP were interpreted as representing liquid contained in pores smaller than approximately 100 nm in diameter. The FSP value is expressed as the dimensionless ratio of the mass of pore water to the mass of dry solids (g g^−1^).

### Pore size determination of the substrates

Average pore sizes were computed by combining estimates of the SSA from NMR spectra with results from FSP measurements. Advantages of this approach is that average pore sizes can be estimated without the need for sample drying and no pore geometry needs to be assumed [19].

Pore size was calculated from the following relation:

$$ 2t=\frac{2\left(\mathrm{F}\mathrm{S}\mathrm{P}\right)}{\sigma {\varphi}_L} $$

where

- 2 *t*
  - average pore size
- FSP
  - fibre saturation point
- *σ*
  - cellulose SSA
- *φ*_L_
  - density of water.

The reported average pore size (2 *t*) is comparable to the average pore diameter.

### Enzymatic hydrolysis experiments

Enzymatic hydrolysis experiments were carried out in 50 mL tubes (TPP Techno Plastic Products AG, Trasadingen, Switzerland) with a 40 mL working volume at 50°C and 30 rpm on an adjustable angle mixing rotator (SB3, Stuart®, Bibby Scientific Limited, Staffordshire, UK). The cellulose concentration was 15 mg mL^−1^, and 50 mM sodium citrate buffer (pH = 4.8) was added. The enzyme mixture was Celluclast 1.5 L and Novozyme 188 (both from Novozymes A/S Bagsvaerd, Denmark) at 5 mg protein per gram cellulose and 500 nkat g^−1^ cellulose, respectively. Novozyme 188 was added to the hydrolysis mixture to compensate for low β-glucosidase activity in Celluclast 1.5 L mixture. Two reaction tubes were withdrawn after 2 days of enzymatic hydrolysis and centrifuged (10 min, 3,000 rcf). Errors in the conversion yields (%) are represented by the minimum and maximum values of two replicates. The supernatant was used to determine the sugar concentration, and the solid residue after two days of hydrolysis was characterized by CP/MAS ^13^C-NMR. The solubilized sugars were quantified using a Dionex ISC-3000 high-performance anion exchange chromatography system coupled to a CarboPac PA1 (250 mm × 4 mm i.d.) column (Dionex, Sweden) and a pulsed amperometric detector (HPAEC-PAD). The conversion of cellulose to glucose was calculated assuming that the theoretical yield of glucose is 1.11 times higher than the weight of cellulose (1.11 g glucose per gram cellulose) due to the addition of water during hydrolysis.

## Additional file

Additional file 1:
**Estimation of inter-particle distance in a sol consisting of cellulose nano-particles in a liquid.**

## Abbreviations

DCr: Degree of crystallinity
FSP: Fibre saturation point
LFAD: Lateral fibril aggregate dimensions
LFD: Lateral fibril dimensions
NCC-OPHS-ND: Nano-crystalline cellulose produced from OPHS-ND pulp substrate
SSA: Specific surface area
OPHS-ND: Oxygen-delignified pre-hydrolysis soda pulp, never-dried
OPHS-D: Oxygen-delignified pre-hydrolysis soda pulp, dried
