# Supplementary material for: Impact of the supramolecular structure of cellulose on the efficiency of enzymatic hydrolysis
Source: Biotechnol Biofuels. 2015 Apr 1;8:56. doi: 10.1186/s13068-015-0236-9 (PMC4394567; doi:10.1186/s13068-015-0236-9)
Supplement: Additional file 1: — Estimation of inter-particle distance in a sol consisting of cellulose nano-particles in a liquid. [file 13068_2015_236_MOESM1_ESM.docx]

**Estimation of inter-particle distance in a sol consisting of cellulose nano-particles in a liquid**

*V_T_* is the total volume of the sol expressed as the sum of the liquid volume (*V_L_*) and the volume of cellulose nano-particles (*V_C_*), equation [1].

$V_{T}=V_{C}+ V_{L}$ [1]

The concentration of the cellulose nano-particles (*K_C_*) expression as mass per volume medium, equation [2], can be recalculated as a cellulose volume based concentration by dividing the cellulose mass (*m_C_*) by the density of the cellulose (*ρ_C_*), equation [3].

$K_{C}=\frac{m_{C}}{V_{T}}$ [2]

$V_{C}=\frac{m_{C}}{\rho_{C}} \leftrightarrow m_{C}=V_{C}\rho_{C}$ [3]

$K_{C}=\frac{m_{C}}{V_{T}}=\frac{V_{C}\rho_{C}}{V_{T}}=\frac{V_{C}\rho_{C}}{V_{C}+V_{L}}$ [4]

Rearranging equation [4] yields an expression for the liquid-to-cellulose volume ratio, equation [5].

$\frac{V_{L}}{V_{C}}=\frac{\rho_{C}}{K_{C}}-1$ [5]

Equation [5] shows correctly that as the concentration of cellulose particles approach the density of cellulose, the liquid volume tends to zero.

To compute an order-of-magnitude estimation of the inter-particle distance between the cellulose nano-particles in a well dispersed reaction medium, a cellulose nano-particle with average dimensions *a* times *a* time *L*, is placed in a liquid filled box of dimensions *A* times *A* times *L*. The cellulose nano-particle is centered laterally in the box and the length axis of the cellulose nano-particle is aligned with the length axis of the surrounding box. The liquid-to-cellulose volume ratio can be expressed in equation [6].

$\frac{V_{L}}{V_{C}}=\frac{\left( A^{2}-a^{2} \right)L}{a^{2}L}=\frac{A^{2}-a^{2}}{a^{2}}$ [6]

Equation [7] combines equation [6] and equation [5].

$\frac{\rho_{C}}{K_{C}}-1=\frac{A^{2}-a^{2}}{a^{2}}$ [7]

Equation [7] can be rearranged to an expression giving the side length of the surrounding box in terms of known entities, equation [8].

$A=a\sqrt{\frac{\rho_{C}}{K_{C}}}$ [8]

An order-of-magnitude estimate of the inter-particle distance (*d*) was calculated as twice the lateral width of the liquid layer surrounding the cellulose nano-particle, equation [9].

$d=2\frac{\left( A-a \right)}{2}=A-a= a\sqrt{\frac{\rho_{C}}{K_{C}}}-a=a\left( \sqrt{\frac{\rho_{C}}{K_{C}}}-1 \right)$ [9]

For a cellulose nano-particle concentration of 15 mg/mL, a cellulose density of 1500 kg/m^3^, and an average cellulose-nano particle width (LFAD) of 20 nm the estimated inter-particle distance was 180 nm. Although the model used is indeed approximate, it shows that the typical inter-particle distances was an order of magnitude larger than the typical enzyme sizes. The conclusion was therefore that no significant hindrance was exerted on the accessibility of enzymes to the cellulose nano-particles due to their density in the reaction medium. In a well dispersed sol of cellulose nano-particles a possible factor limiting the effective rate of enzymatic conversion could be the amount of specific surface area.
